# Validation of Monocular Pupillometry in Healthy Controls and Patients With Autonomic Dysfunction: Pupillary Biomarkers for Autonomic Failure

**DOI:** 10.1111/ene.70320

**Published:** 2025-08-19

**Authors:** Laura Sander, Grace Oommen, Conor Brophy, Silver Bohus‐Roper, Giacomo Chiaro, Fion Bremner, Valeria Iodice

**Affiliations:** ^1^ Autonomic Unit The National Hospital for Neurology and Neurosurgery London UK; ^2^ Department of Brain Repair and Rehabilitation University College London Queen Square Institute of Neurology London UK; ^3^ Neurologic Clinic and Policlinic, Department of Medicine and Clinical Research University Hospital Basel and University of Basel Basel Switzerland; ^4^ Department of Neuro‐Ophthalmology The National Hospital for Neurology and Neurosurgery London UK

**Keywords:** apraclonidine, autoimmune autonomic ganglionopathy, autonomic dysfunction, neurodegenerative diseases, pupillometry

## Abstract

**Background:**

Pupillary function is frequently impaired in autonomic disorders, and biomarkers for early diagnosis and disease progression are urgently needed. Pupillometry allows for noninvasive ocular autonomic evaluation. This prospective study technically and clinically validates a handheld monocular pupillometer available for broad application as an autonomic screening tool in autonomic disorders.

**Methods:**

A total of 40 controls and 100 patients with autonomic disorders underwent pupillometry using the PLR‐4000(NeurOptics). Pupillary parasympathetic and sympathetic function were assessed by responses to a light stimulus and to 0.5% apraclonidine eye drops, respectively. Test–retest assessments and validations against a binocular device were performed.

**Results:**

In healthy controls, the mean light reflex ratio was 42% ± 5.7% and the median response to apraclonidine was −5.0% (−8.8%–2.8%). Monocular and binocular pupillometers presented similar results. Test–retest experiments showed: median light response difference 3.0% (1.0%–4.8%), median % difference in response to apraclonidine 5.2% (2.2%–10.6%). In patients with neurodegenerative disorders (*n* = 24), autonomic neuropathies (*n* = 39), and autonomic ganglionopathies (*n* = 9), pupillary abnormalities were very prevalent (52%, 45%, and 100%, respectively). All patients with intermittent autonomic disorders had normal pupillomotor function.

**Conclusions:**

The presented device provides accurate, reproducible assessments of pupillary autonomic function in healthy controls and patients with autonomic disorders. With normative data provided, it is an easily accessible, well‐tolerated tool to quantitatively assess pupillomotor innervation in a broad clinical setting. Further studies are warranted to explore its potential as a noninvasive biomarker, complementing standard autonomic function tests for early detection, monitoring disease progression, and evaluating treatment response in disorders with autonomic failure.

## Graphical abstract

This prospective study introduces normative parasympathetic and sympathetic ocular data of 40 healthy controls and validates the monocular handheld pupillometer PLR‐4000 (NeurOptics) in 100 patients with autonomic dysfunction. We present detailed protocols including the use of pharmacologic stimuli as an easily accessible, noninvasive tool for pupillomotor evaluation in a broad clinical and scientific setting.

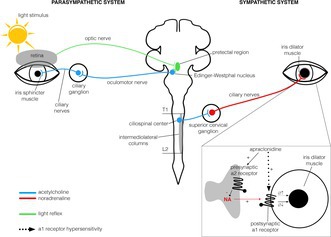

## 1 Introduction

Autonomic manifestations including cardiovascular, gastrointestinal, urogenital, thermoregulatory, secretomotor, or pupillomotor dysfunction are prevalent and disabling features in various disorders affecting the central and peripheral nervous system. Early autonomic impairment can present the first symptom, for example, in alpha‐synucleinopathies such as multiple system atrophy (MSA) [1] and in autonomic neuropathies such as hereditary transthyretin amyloidosis (ATTRv) [2]. Cardiovascular autonomic failure is a key cause of increased morbidity and mortality in patients with Parkinson's disease (PD) [3], diabetes mellitus [4], and Guillain–Barré Syndrome [5]. Evaluation of autonomic dysfunction can assist with early diagnoses in prodromal disease stages such as in prediabetes [6] or asymptomatic TTR variant carriers [2]. It can also be useful in differential diagnoses of alpha‐synucleinopathies [7, 8].

To date, the search for widely available, noninvasive biomarkers in neurodegenerative diseases and disorders amenable to disease‐modifying treatments remains a critical priority. Autonomic dysfunction is commonly assessed using a battery of validated quantitative measures, primarily focused on evaluating cardiovascular and sudomotor function [9]. However, standardized autonomic function testing is still not widely available [10]. Of note, pupillomotor dysfunction is also prevalent in many autonomic disorders [11, 12], serving as a potential disease signature in conditions such as autoimmune autonomic ganglionopathy (AAG) [13, 14]. Hence, pupillometry may provide a valuable additional tool for assessing autonomic involvement across multiple domains and different entities. In contrast to diseases with autonomic failure which frequently affect pupillary function [11], intermittent autonomic disorders including postural tachycardia syndrome (POTS), initial orthostatic hypotension (iOH) or autonomically mediated syncope typically do not present with persistent and widespread autonomic impairment. These conditions would therefore not be expected to show autonomic pupillary impairment.

**FIGURE 1:**
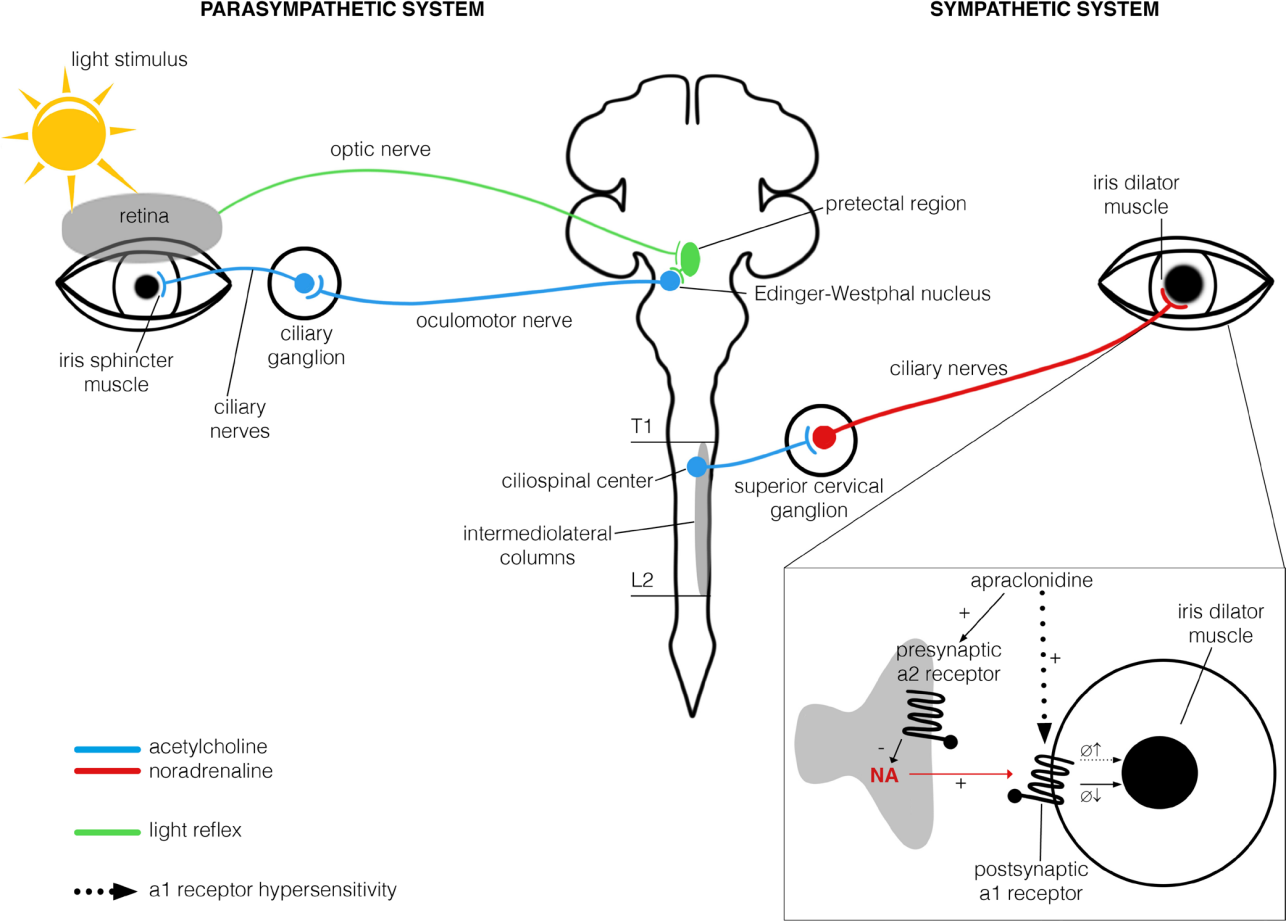
Illustration of pupillary parasympathetic (left) and sympathetic (right) innervation, light reflex, and effects of apraclonidine. Parasympathetic preganglionic neurons originate in the mesencephalon and project via the oculomotor nerve to the ciliary ganglion. Postganglionic fibers from the ciliary ganglion innervate the iris sphincter muscle, mediating pupil constriction and accommodation reflex. Acetylcholine serves as a neurotransmitter at both pre‐ and postganglionic synapses. In contrast, sympathetic preganglionic neurons arise from the thoracic spinal cord and project to the superior cervical ganglion, also using acetylcholine. Postganglionic sympathetic fibers then innervate the iris dilator muscle, with noradrenaline acting as the neurotransmitter to mediate pupil dilation. The zoomed‐in image illustrates the effects of apraclonidine. In a healthy pupil (plain arrow) apraclonidine primarily stimulates presynaptic α2‐receptors, inhibiting noradrenaline release and reducing activation of the iris dilator muscle, which usually provokes a mild miosis. In a sympathetically denervated pupil with upregulation of α1‐receptors, apraclonidine directly stimulates postsynaptic α1‐receptors (dotted arrow), resulting in a pupillary mydriasis.

Parasympathetic ocular activation results in pupillary constriction and accommodation (Figure 1) with parasympathetic dysfunction potentially causing light sensitivity or troubles with focusing. Sympathetic function mediates mydriasis (Figure 1), and denervation can present with difficulties adjusting to dim light conditions.

Pupillometry provides quantitative measurements of both parasympathetic (via responses to light) and sympathetic (using pharmacologic stimuli with apraclonidine; Figure 1) ocular function. Apraclonidine was applied previously in the detection of Horner syndrome [15, 16] and to reveal subtle pupillary sympathetic denervation in patients with migraine [17], but it has not been widely used in patients with autonomic dysfunction for the assessment of ocular sympathetic innervation so far.

Currently, the standard pupillomotor assessment relies on advanced binocular pupillometry allowing for exclusion of distractive light and simultaneous measurements of both pupils, but its clinical use has been scarce and restricted to specialized centers only. Monocular handheld pupillometry with use of apraclonidine eye drops offers an accessible, scalable solution at lower cost for integrating autonomic screening into clinical care of patients and expanding its use in general neurology including bedside assessments.

The objectives of this prospective study were (1) to assess parasympathetic and sympathetic pupillary function in healthy individuals in order to generate normative data, (2) to technically validate dynamic monocular pupillometry using PLR‐4000 (NeurOptics) against a previously published binocular device [16], and (3) to clinically assess pupillary function in patients with autonomic failure and intermittent autonomic disorders.

## 2 Methods

### 2.1 Study Population

In total, 100 patients and 40 healthy controls were assessed prospectively at the national tertiary referral Autonomic Centre, National Hospital for Neurology and Neurosurgery, London, UK, from April–December 2024 using a standard battery of pupillometric evaluations. Patients with autonomic dysfunction underwent pupillometry as part of their routine autonomic assessment.

Individuals receiving systemic or topical medical treatment likely to interfere with pupillary function were excluded from the study. Controls were included when no eye history other than refractory errors and no disease affecting pupillary function (including migraine or diabetes) were present. Consecutive patients with a broad range of autonomic disorders were included and divided into four groups:(1) Neurodegenerative disorders including alpha‐synucleinopathies (pure autonomic failure (PAF), MSA, PD, dementia with Lewy bodies) as well as fatal familial insomnia, Triple‐A syndrome, and cerebellar ataxia, neuropathy, and vestibular areflexia syndrome (CANVAS);(2) autonomic neuropathies due to diabetes mellitus, hereditary transthyretin amyloidosis (ATTRv), or amyloid light chain (AL) amyloidosis;(3) autonomic ganglionopathies including AAG, Ross, or Holmes‐Adie syndromes [18];(4) autonomic intermittent disorders (POTS, iOH, autonomically mediated syncope).

Ethics approval was obtained by national (London Harrow Research Ethics Committee) and institutional (University College London and Imperial College London) research authorities (no. 197553). The study conforms to World Medical Association Declaration of Helsinki and was conducted according to the Essential Items for Reporting Diagnostic Accuracy Studies (STARD 2015) [19]. Pupillary assessments were undertaken with the understanding and written consent of each participant.

### 2.2 Pupillometry

Study participants underwent pupillometry in a dark room, facing the clinician. The infrared pupillometer (PLR‐4000, NeurOptics) was placed directly over each eye, displaying the whole pupil (Video S1). Pupil diameters (mm) and their responses to an automated light stimulus were recorded by monocular assessment for each eye. Measurements were made of the reflex pupillary constriction to a standard 1 s white light stimulus (180 μW, pulse onset after 0.2 s, measurement duration 8.0 s; Video S2) and of the resting pupil diameter averaged over 5 s in complete darkness before (Video S3 A/C) and 40–60 min after (Video S3 B/D) application of 0.5% apraclonidine eye drops. Parasympathetic ocular function was quantified by relative pupillary constriction to the light stimulus (reflex amplitude/starting diameter expressed as %) and maximal constriction velocity (mm/s). Ocular sympathetic dysfunction was assessed by measuring the redilation time after cessation of the light stimulus (T75, defined as the time taken by the pupil to recover 75% of the initial resting pupil size following the light reaction) and measuring the change in resting pupil diameter 40–60 min after application of 0.5% apraclonidine eye drops [16].

A subset of healthy individuals underwent test–retest experiments for light reflex as well as repeated assessments every 5 min during the 40–60 min apraclonidine interval aimed at evaluation of greatest apraclonidine effect.

In patients with autoimmune autonomic ganglionopathy (AAG), pupillary fatigue [13] was also assessed with a 2 s light stimulus (180 μW, pulse onset after 0.2 s, measurement duration 8.0 s; Video S4).

### 2.3 Validation Protocol in Binocular Pupillometry

In addition to the protocol described above, 20 individuals were evaluated on the same day with a binocular pupillometer (DP2000 NeurOptics). Pupillary light reflex assessed by the average reflex constriction to three repetitions of a standard 1 s light stimulus as well as resting pupil diameter (averaged over 3 s) in complete darkness before and after topical application of 0.5% apraclonidine was evaluated as previously described [16].

### 2.4 Statistical Analyses

The pupil response to light (light reflex ratio) was calculated as (diameter dark–diameter light)/diameter dark (expressed as %). The apraclonidine effect was expressed as (diameter after–diameter before)/diameter before eyedrops (in %), and assessed after 40 min and 60 min, with negative values indicating miotic response and positive values corresponding to mydriatic response.

Continuous variables were reported as mean ± SD (normally distributed data) or median (IQR) for non‐normally distributed data. Nominal data were expressed as number or percentage.

Statistical analyses were performed using SPSS 29 (Armonk, NY: IBM Corp)

## 3 Results

### 3.1 Technical Validation

#### 3.1.1 Normative Data in Healthy Controls

Tolerance of pupillometric assessments and apraclonidine eye drops were good in general. Median age of healthy subjects at time of testing was 36.5 (30.0–47.3) years; 53% were females.

Of 40 controls, light reflex ratio could be assessed in 77 eyes, and effect of apraclonidine in 80 eyes. Pupillary data of healthy controls are summarized in Table 1. Mean light reflex ratio was 42% ± 5.7% and median response to apraclonidine was −5.0 (−8.8–2.8) %.

**TABLE 1:** Pupillary data in healthy controls.

Variable	Number (eyes)	Mean [± SD] Median [IQR]	Range
Dark diameter [mm]	77	6.7 [5.8–7.1]	4.2; 8.0
Light diameter [mm]	77	3.9 [3.2–4.4]	2.3; 5.2
Light reflex ratio [%]	77	42 [± 5.7]	28; 58
Average constriction velocity [mm/s]	77	−2.9 [± 0.5]	−3.9; −2.0
Maximum constriction velocity [mm/s]	77	−4.9 [± 0.8]	−6.6; −3.5
Average dilation velocity [mm/s]	75	1.2 [± 0.3]	0.6; 1.9
T75	69	4.2 [± 1.1]	2.3; 6.3
Apraclonidine effect [%]	80	−5.0 [−8.8–2.8]	−17; 15.8
Apraclonidine difference [mm]	80	−0.3 [−0.6–0.2]	−1.22; 0.9

In healthy subjects, the median intra‐individual difference in pupillary resting size between both pupils was 0.2 (0.1–0.4) mm. In the following, significant anisocoria was defined as the intra‐individual difference in pupillary resting size > 0.7 mm (mean + 1.96*SD).

Median intra‐individual difference in light reflex ratio was 2.5 (1.0–4.0) % between both eyes. Mean intra‐individual difference of effect to apraclonidine was −3.3% ± 5.4% (when considering the biggest difference at 40 min or 60 min interval). A miotic response to apraclonidine was found in 51 eyes, whereas there was a mild mydriatic response in 29 eyes at 40 min (first assessment). Exemplary normal results for light reflex and response to apraclonidine are shown in Video S2A and Video S3A/C, respectively.

Younger controls (below the median of 30 years) had significantly larger mean resting pupils compared to older individuals (*p* = 0.001, 95% CI = 0.4–1.4), but there was no difference in mean light response (*p* = 0.355). There was no statistical difference in mean resting pupillary size and mean relative pupillary constriction to light between men and women.

#### 3.1.2 Validation With Binocular Pupillometer

Thirteen patients and seven healthy controls underwent pupillary assessment with both the monocular PLR‐4000 and the binocular DP2000. Results were comparable as assessed by two different pupillometers: five patients had no ocular autonomic dysfunction, one patient with Holmes‐Adie syndrome presented with parasympathetic denervation, five patients had isolated sympathetic dysfunction (including one patient with ATTRv whose sympathetic response could not be evaluated on the DP2000 due to repeat blinking), and two patients had combined parasympathetic and sympathetic denervation. In one of the latter, parasympathetic impairment assessed by PLR4000 was only detectable by mildly impaired constriction velocities, showing normal relative pupillary constriction. All healthy controls had normal findings as assessed by both pupillometers.

#### 3.1.3 Intra‐Individual Test–Retest Variability

Twenty healthy individuals (a total of 40 eyes) underwent test–retest experiments. Median intra‐individual difference in resting pupil size was 0.4 (0.1–0.7) mm. Median light response difference was 3.0% (1.0%–4.8%). Median % difference in response to apraclonidine (40 min) was 5.2% (2.2%–10.6%).

#### 3.1.4 Effect of Apraclonidine

Twelve controls had repeat assessments every 5 min during the 40–60 min interval after topical application of apraclonidine. The largest differences in pupillary size compared to measurements before use of eye drops were found at 40 min (in 13 eyes), 45 min (1 eye), 50 min (2 eyes), 55 min (2 eyes), and 60 min (6 eyes).

### 3.2 Clinical Validation

In total, 100 patients with autonomic symptoms due to different etiologies underwent pupillary assessment. Median age at time of testing was 58 (43.75–66.25) years; 50% were males.

#### 3.2.1 Disorders Presenting With Neurodegeneration

In patients with alpha‐synucleinopathies (*n* = 21; 11 PAF, 7 MSA, 2 PD, 1 dementia with Lewy bodies), a group of adult‐onset and progressive neurodegenerative disorders frequently presenting with autonomic failure, pupillary abnormalities were very common in 11/21 (52%). The most prevalent pupillary abnormality was sympathetic denervation in 9/21 (43%). One patient with PAF had bilaterally reduced constriction velocities and borderline light response indicating parasympathetic impairment, and one patient with MSA presented with anisocoria, but normal sympathetic and parasympathetic assessment.

Of the patients with sympathetic ocular dysfunction, seven presented with bilateral and two with unilateral involvement. Six individuals had PAF (of a total of 11 patients assessed with PAF), two had MSA (2/7), and one had dementia with Lewy bodies (1/1).

Normal results were found in 4/11 patients with PAF, 4/7 patients with MSA, and 2/2 PD patients.

One patient with fatal familial insomnia and recent onset of symptoms presented with normal pupillometry results.

Borderline unilateral sympathetic impairment was found in one patient with Triple‐A syndrome.

One patient with CANVAS showed isolated parasympathetic impairment.

#### 3.2.2 Autonomic Neuropathies

In individuals with diabetes mellitus (*n* = 7) and hereditary (ATTRv, *n* = 29) or AL amyloidosis (*n* = 2), all commonly presenting with autonomic neuropathy, pathologic results were found in 17/38 (45%). Seven patients had severe combined sympathetic and parasympathetic ocular denervation (four patients with ATTRv and three patients with diabetes). Ten patients (8 with ATTRv, 1 with AL amyloidosis, 1 with diabetes) had sympathetic impairment. Sympathetic dysfunction was more severe in patients with combined denervation compared to patients with isolated sympathetic dysfunction (mean mydriasis of 62% versus 28%, *p* < 0.001). Videos S2B and S3B/D show exemplary pupillary parasympathetic and sympathetic assessments in a diabetic patient with combined denervation.

One patient with baroreflex failure due to former neck radiation for lymphoma treatment presented with mild isolated unilateral sympathetic denervation.

21 individuals had normal pupillary assessment: 17/29 with ATTRv, 3/7 with diabetes, and 1/2 with AL amyloidosis.

#### 3.2.3 Autonomic Ganglionopathies

All nine patients with autonomic ganglionopathies (100%) including six patients with AAG (five seropositive, one seronegative) as well as three patients with Ross/Holmes‐Adie syndrome, had abnormal pupillary assessments. While all patients with Ross/Holmes‐Adie syndrome had isolated parasympathetic impairment, pupillary autonomic profiles were mixed in AAG: One patient (seropositive AAG receiving immunomodulatory treatment) had isolated parasympathetic impairment as well; three patients had sympathetic dysfunction (two seropositive with immunomodulatory treatment, one with seronegative AAG), and two patients (both with seropositive AAG and immunomodulatory treatment) had combined denervation. One patient with bilateral sympathetic impairment (seronegative AAG) had marked anisocoria.

#### 3.2.4 Pupillary Fatigue

**FIGURE 2:**
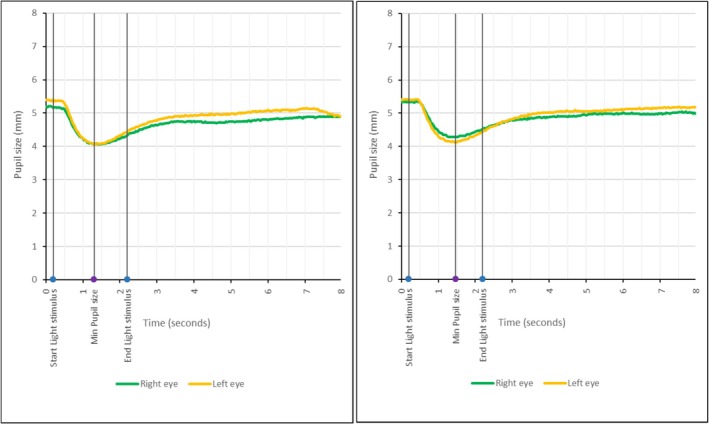
Pupillary fatigue assessment in a patient with autoimmune autonomic ganglionopathy. Before treatment with intravenous immunoglobulins (left), pupillary escape starts at 0.97 s before the end of the light stimulus, improving to 0.74 s after treatment (right).

Pupillary fatigue, a pupillary signature in patients with seropositive AAG, was assessed in six patients with AAG (5 seropositive, 1 seronegative). Three of them (all seropositive) presented with non‐sustained miosis to the 2 s light stimulus (Video S4B). The three patients without pupillary fatigue (2 seropositive, 1 seronegative) had previous immunomodulatory treatment. One patient underwent pupillometry before and after treatment with intravenous immunoglobulins. Although marked parasympathetic impairment and borderline mydriatic response to apraclonidine eye drops were comparable before and after treatment, pupillary fatigue seemed to improve mildly after treatment: the time at which pupillary escape (redilation) started with respect to the end of the light stimulus was 0.74 s after treatment compared to 0.97 s before treatment (Figure 2).

#### 3.2.5 Intermittent Autonomic Disorders

All patients with intermittent autonomic disorders (*n* = 28; 6 patients with autonomically mediated syncope, 1 patient with POTS, 13 patients with iOH, and 8 patients with a combination of the previously mentioned) had unremarkable pupillary assessment. Of the latter, two patients had asymptomatic mild anisocoria.

## 4 Discussion

Autonomic function testing is essential to confirm and quantify the degree of autonomic dysfunction and can help to differentiate between Parkinsonian syndromes [20]. Ocular autonomic assessment using pupillometry is an additional screening tool for the evaluation of the autonomic nervous system. This study has technically and clinically validated the dynamic handheld monocular pupillometer PLR‐4000 (NeurOptics) and provides normative data for clinical use.

Our healthy cohort data are in line with previous studies showing a larger pupillary size with younger age [11, 21]. Healthy individuals presented a 28%–58% relative pupillary constriction to light, comparable to previous assessments on different devices [11].

Monocular and binocular [16] devices had similar qualitative findings for normal and pathologic results, yielding high accuracy in 20 individuals. A previous study found better repeatability and agreement in measuring pupil size using a binocular pupillometer compared to a handheld device [22]. However, comparison of monocular and binocular infrared pupillometers showed similar pupil diameters under mesopic conditions [23].

The PLR‐4000 presented high test–retest repeatability for responses to light and apraclonidine in 20 healthy subjects, offering two consistent and robust outcome measures that can be useful for follow‐up and response to treatment assessments. However, resting pupil diameter, which is highly sensitive to arousal and emotions [24], showed higher variability and should therefore not be used as an outcome measure.

Our data support the use of apraclonidine eye drops for quantitative sympathetic pupillomotor assessment, as previously described [15]. Our healthy control data are comparable to the data assessed by the previously published validation protocol [16] where healthy eyes showed a mean reduction of −0.44 mm (equivalent to a relative 7% decrease), with measured differences ranging from −1.3 to +0.8 mm (−20 to +16%). We therefore defined a pathologic cut‐off at 16% mydriatic response. Our data found time differences in response to apraclonidine that might be due to factors such as individual metabolism or local penetration which can be affected by ocular surface disease such as dry eyes. Hence, we suggest a first assessment after 40 min and in case of no clear response, repeat testing after a total of 60 min.

In this cohort, controls (median age 36.5 years) were younger than patients (median age 58.0 years), their age ranges were similar (22–79 years and 21–81 years, respectively). Younger controls had larger resting pupil size than older individuals, but responses to light and apraclonidine were not age‐dependent, reason why we think that the presented normative data for parasympathetic and sympathetic autonomic assessment can be applied for all age ranges.

In the subset of patients with autonomic neuropathies, sympathetic dysfunction was more severe in patients with combined sympathetic and parasympathetic denervation, likely presenting a more advanced stage of disease compared to patients with isolated sympathetic dysfunction, reflecting milder autonomic involvement. Greater sympathetic denervation appears to produce α1‐receptor supersensitivity to apraclonidine, resulting in more pronounced mydriasis.

In patients with a broad range of autonomic disorders, pupillometry including pharmacological stimulation was able to detect abnormal pupillomotor function, while pupillometric results in the subgroup with intermittent autonomic disorders were normal. This is in line with the nature of these diseases, which do not present with persistent autonomic failure. In patients with POTS, a recent study found a larger pupil size during head‐up tilt compared to the supine position, which was not found in healthy controls [25]. This finding was considered due to sympathetic overactivity. We did not measure pupil size in both supine and tilted positions as this was not the purpose of our study. However, the study by Rodriguez et al. did not find any significant positional changes in constriction velocities in patients with POTS and in healthy controls, and the latter is in line with our results presenting normal constriction velocities in POleftTS patients.

Despite small sample sizes in our cohort and similar to previously published results [11], patients with PAF frequently had pupillary abnormalities, with sympathetic denervation being the most common, while pupillomotor dysfunction was less prevalent in patients with MSA. Using a former device by the same manufacturer to assess response to light reflex, a previous study described a correlation of constriction velocities with severity of MSA‐specific symptoms and of constriction velocities and average dilation velocity with autonomic symptom severity [26].

We found frequent, mainly sympathetic or mixed sympathetic and parasympathetic dysfunction in individuals with ATTRv. A previous study reported impaired parasympathetic and sympathetic parameters in a larger cohort of 32 ATTRv patients [27]. Pupillary involvement is equally known to be a prevalent feature in diabetic patients [28] and can be present before cardiovascular autonomic changes [29].

All patients with autonomic ganglionopathies presented with abnormal pupillary assessments, resulting in a higher prevalence of pupillary dysfunction than in patients with autonomic neuropathies and neurodegenerative diseases. Pupillary fatigue, defined as a premature redilation of the pupil to a sustained light impulse, is a hallmark in seropositive AAG [13, 14]. Pupillary fatigue was observed in three of our patients with seropositive AAG, establishing a signature test that helps with early diagnosis and monitoring treatment response.

In conclusion, monocular pupillometry with the presented protocols, including the application of 0.5% apraclonidine using the handheld PLR‐4000 device, is a well‐tolerated, easily accessible tool for accurate and robust measurements of pupillomotor function, which can also be performed by non‐ophthalmologists and at the bedside. Our presented data, including normative values from healthy controls, may enhance its use in clinical settings, allowing for the confirmation and quantification of autonomic dysfunction in patients with neurodegenerative diseases. Further studies are needed to evaluate whether monocular pupillometry can be used as a tool for early autonomic screening, monitoring disease progression, and response to treatment in different disorders.

## Conflicts of Interest

L.S. is supported by the University of Basel, Switzerland, and the Freiwillige Akademische Gesellschaft Basel. G.O., C.B., S.B., G.C., F.B. report no disclosures. V.I. is supported by the National Institute for Health Research, University College London Hospitals Biomedical Research Centre and by the Autonomic Charitable Trust (ACT) (Lord Bagri) Research Award. V.I. has received honoraria from Theravance Biopharma not related to this work.

## Supporting information


**Video S1:** Educational video showing how to perform pupillometry with a monocular, handheld device.
**Video S2:** Parasympathetic pupillary assessment in a healthy individual (A) and a patient with diabetes (B). The healthy control shows normal miotic response to light stimulus, whereas the patient presents with reduced resting pupil size, impaired light reflex, and irregular pupil shape.
**Video S3:** Resting pupil diameter assessment before (A/B) and after (C/D) application of apraclonidine in a healthy individual (A/C) and a patient with diabetes (B/D). While there is a normal miotic response in the healthy eye, the patient presents a mydriatic response to apraclonidine indicating sympathetic denervation.
**Video S4:** Pupillary fatigue assessments in a healthy individual (A) and a patient with autoimmune autonomic ganglionopathy (B). The healthy control presents with sustained pupillary constriction during a 2 s light stimulus, while the patient shows premature pupillary escape before the end of the light stimulus.

## Data Availability

The data that support the findings of this study are available on request from the corresponding author. The data are not publicly available due to privacy or ethical restrictions.
