# Supplementary figures and images for: Validation of Monocular Pupillometry in Healthy Controls and Patients With Autonomic Dysfunction: Pupillary Biomarkers for Autonomic Failure
Source: Eur J Neurol. 2025 Aug 19;32(8):e70320. doi: 10.1111/ene.70320 (PMC12365000; doi:10.1111/ene.70320)

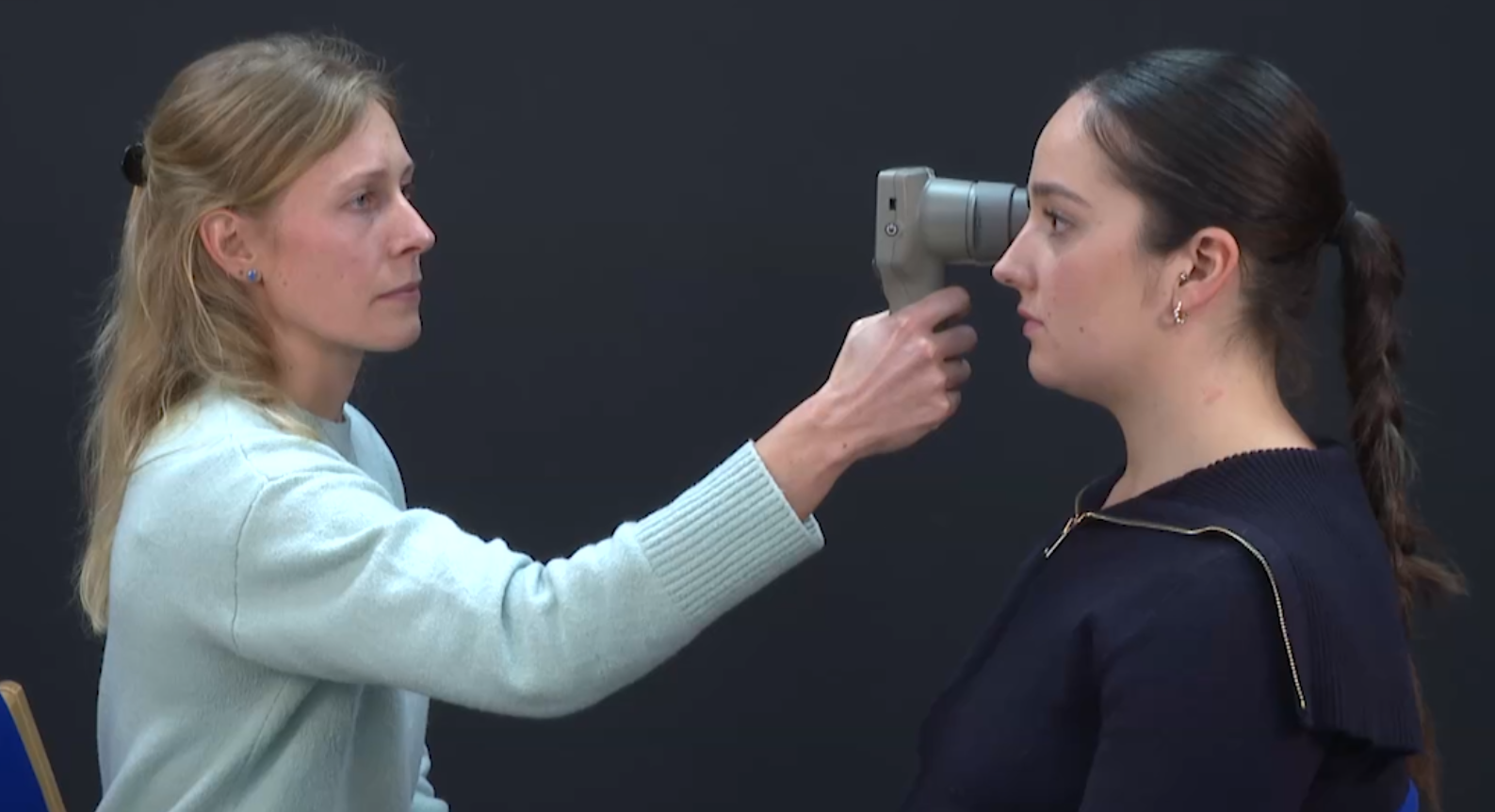

Supplement: Supplementary file 1 — Video S1: Educational video showing how to perform pupillometry with a monocular, handheld device. Video S2: Parasympathetic pupillary assessment in a healthy individual (A) and a patient with diabetes (B). The healthy control shows normal miotic response to light stimulus, whereas the patient presents with reduced resting pupil size, impaired light reflex, and irregular pupil shape. Video S3: Resting pupil diameter assessment before (A/B) and after (C/D) application of apraclonidine in a healthy individual (A/C) and a patient with diabetes (B/D). While there is a normal miotic response in the healthy eye, the patient presents a mydriatic response to apraclonidine indicating sympathetic denervation. Video S4: Pupillary fatigue assessments in a healthy individual (A) and a patient with autoimmune autonomic ganglionopathy (B). The healthy control presents with sustained pupillary constriction during a 2 s light stimulus, while the patient shows premature pupillary escape before the end of the light stimulus. [file ENE-32-e70320-s001.zip › 1.Placeholder_EducationalVideo.PNG]

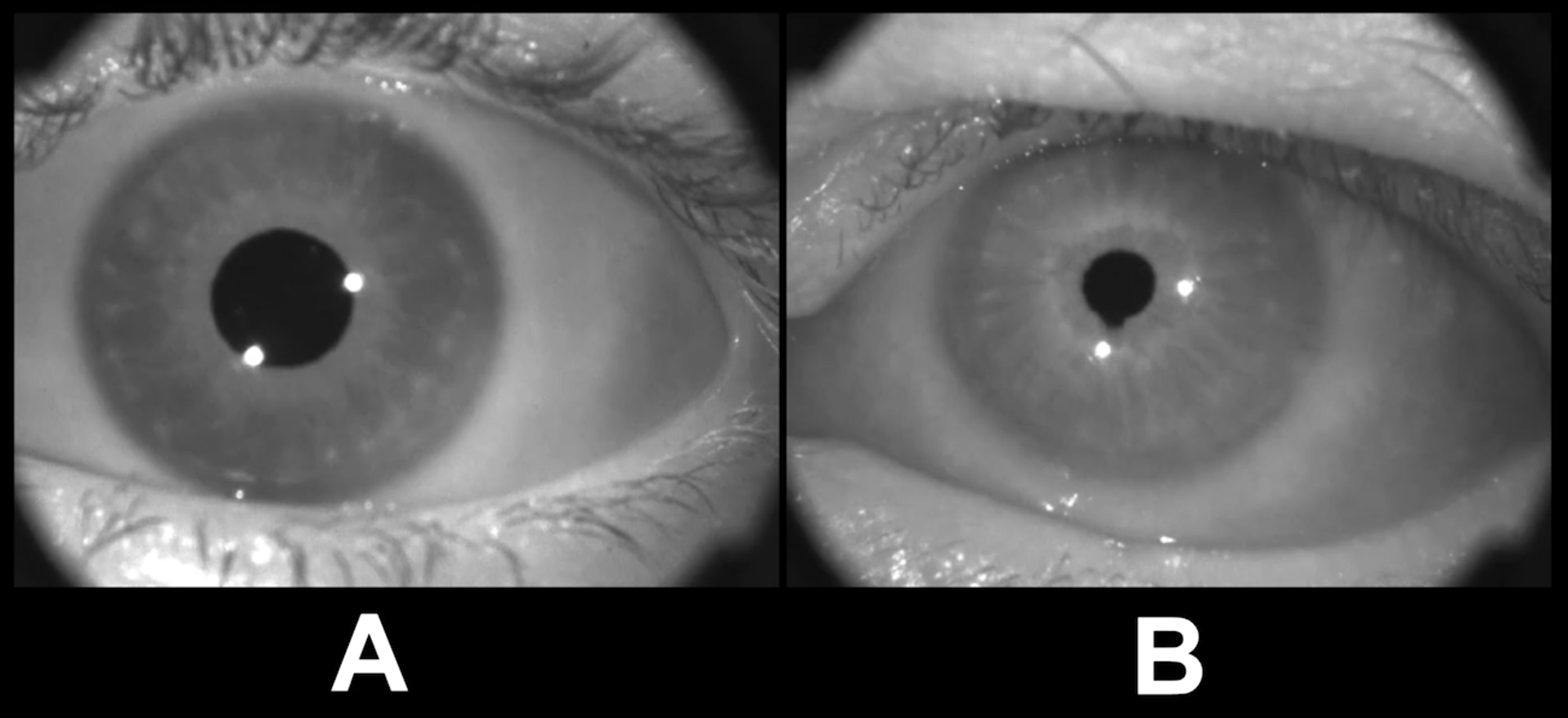

Supplement: Supplementary file 1 — Video S1: Educational video showing how to perform pupillometry with a monocular, handheld device. Video S2: Parasympathetic pupillary assessment in a healthy individual (A) and a patient with diabetes (B). The healthy control shows normal miotic response to light stimulus, whereas the patient presents with reduced resting pupil size, impaired light reflex, and irregular pupil shape. Video S3: Resting pupil diameter assessment before (A/B) and after (C/D) application of apraclonidine in a healthy individual (A/C) and a patient with diabetes (B/D). While there is a normal miotic response in the healthy eye, the patient presents a mydriatic response to apraclonidine indicating sympathetic denervation. Video S4: Pupillary fatigue assessments in a healthy individual (A) and a patient with autoimmune autonomic ganglionopathy (B). The healthy control presents with sustained pupillary constriction during a 2 s light stimulus, while the patient shows premature pupillary escape before the end of the light stimulus. [file ENE-32-e70320-s001.zip › 2.Placeholder_LightReaction.PNG]

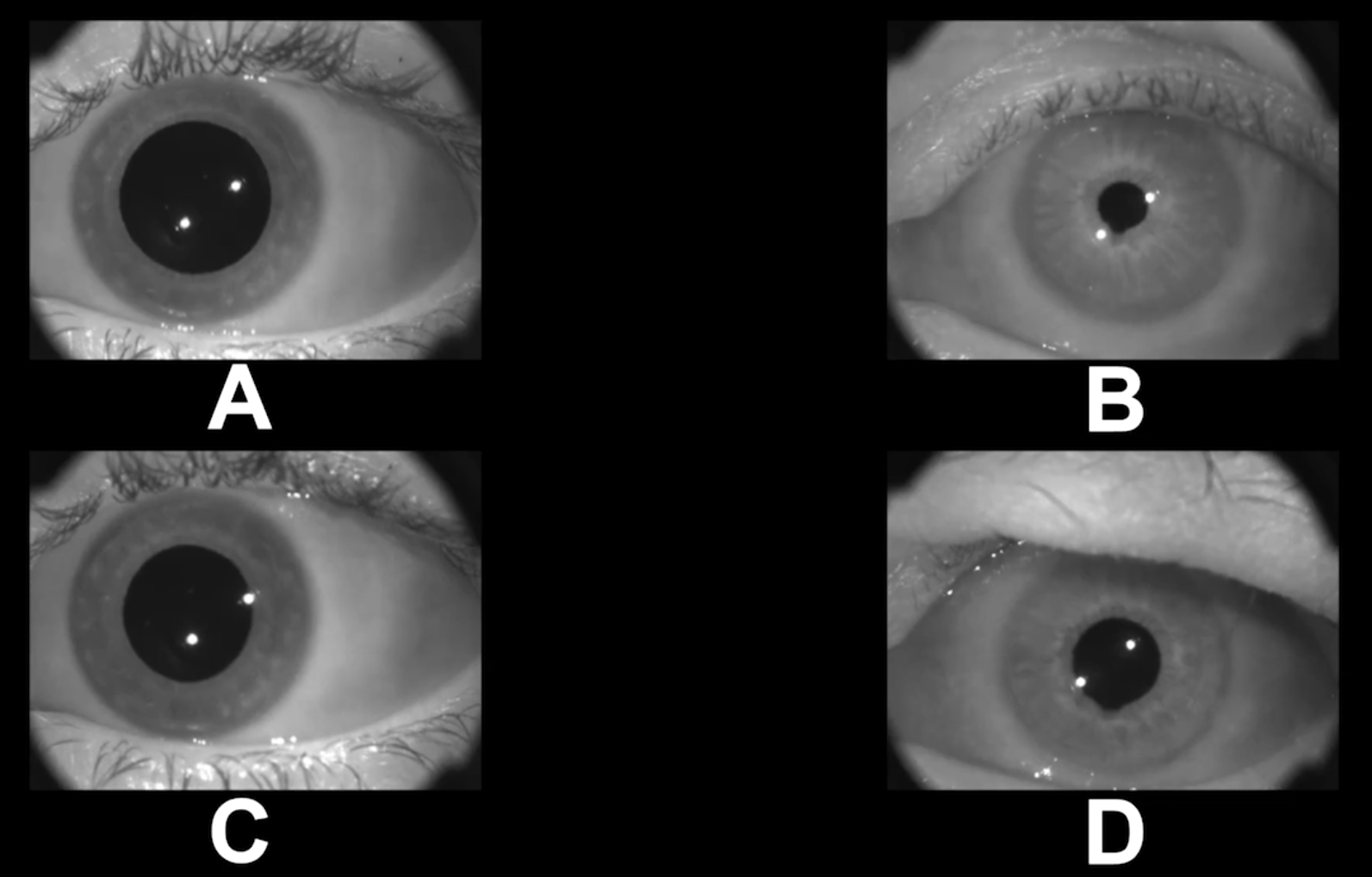

Supplement: Supplementary file 1 — Video S1: Educational video showing how to perform pupillometry with a monocular, handheld device. Video S2: Parasympathetic pupillary assessment in a healthy individual (A) and a patient with diabetes (B). The healthy control shows normal miotic response to light stimulus, whereas the patient presents with reduced resting pupil size, impaired light reflex, and irregular pupil shape. Video S3: Resting pupil diameter assessment before (A/B) and after (C/D) application of apraclonidine in a healthy individual (A/C) and a patient with diabetes (B/D). While there is a normal miotic response in the healthy eye, the patient presents a mydriatic response to apraclonidine indicating sympathetic denervation. Video S4: Pupillary fatigue assessments in a healthy individual (A) and a patient with autoimmune autonomic ganglionopathy (B). The healthy control presents with sustained pupillary constriction during a 2 s light stimulus, while the patient shows premature pupillary escape before the end of the light stimulus. [file ENE-32-e70320-s001.zip › 3.Placeholder_Apraclonidine.PNG]

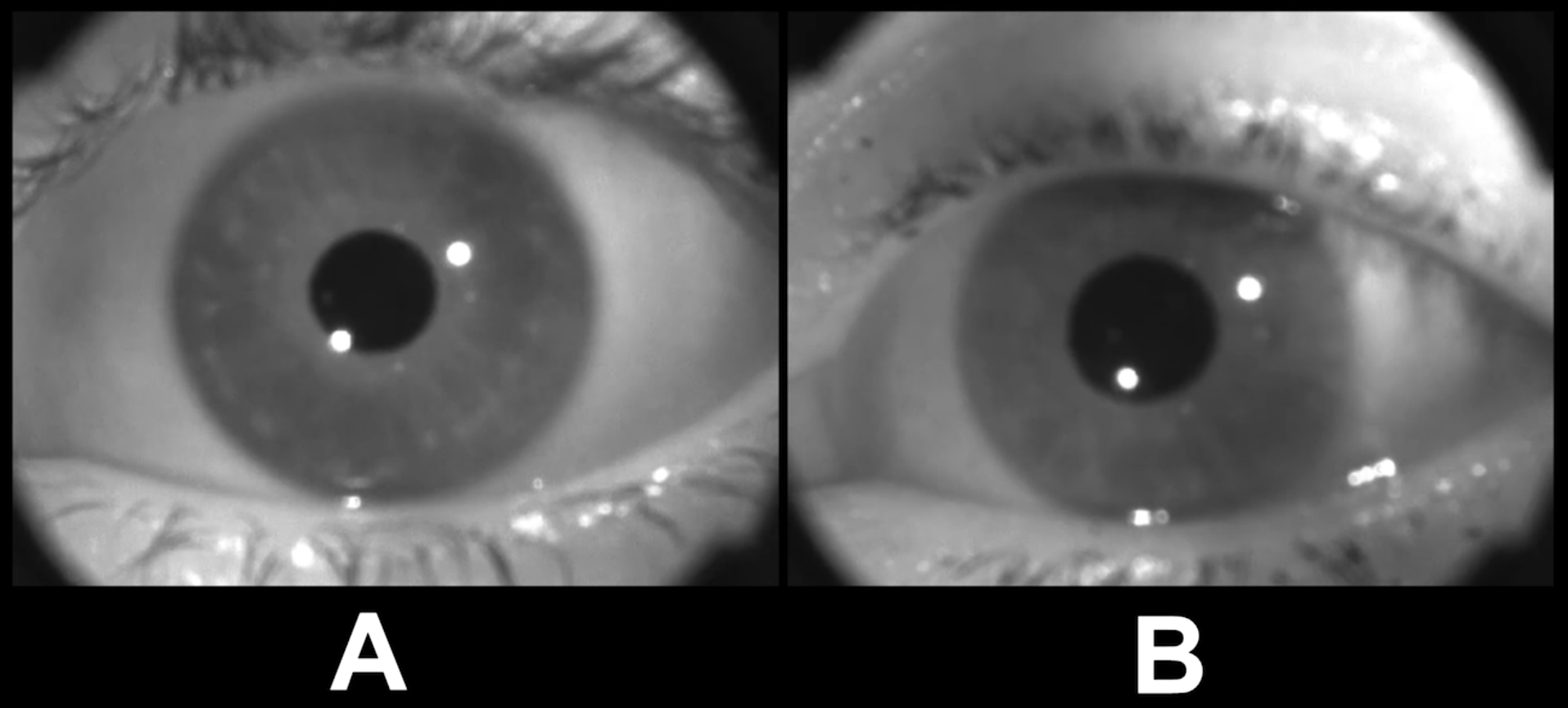

Supplement: Supplementary file 1 — Video S1: Educational video showing how to perform pupillometry with a monocular, handheld device. Video S2: Parasympathetic pupillary assessment in a healthy individual (A) and a patient with diabetes (B). The healthy control shows normal miotic response to light stimulus, whereas the patient presents with reduced resting pupil size, impaired light reflex, and irregular pupil shape. Video S3: Resting pupil diameter assessment before (A/B) and after (C/D) application of apraclonidine in a healthy individual (A/C) and a patient with diabetes (B/D). While there is a normal miotic response in the healthy eye, the patient presents a mydriatic response to apraclonidine indicating sympathetic denervation. Video S4: Pupillary fatigue assessments in a healthy individual (A) and a patient with autoimmune autonomic ganglionopathy (B). The healthy control presents with sustained pupillary constriction during a 2 s light stimulus, while the patient shows premature pupillary escape before the end of the light stimulus. [file ENE-32-e70320-s001.zip › 4.Placeholder_Fatigue.PNG]
